# Differential localisation of BPIFA1 (SPLUNC1) and BPIFB1 (LPLUNC1) in the nasal and oral cavities of mice

**DOI:** 10.1007/s00441-012-1490-9

**Published:** 2012-09-18

**Authors:** Maslinda Musa, Kirsty Wilson, Le Sun, Apoorva Mulay, Lynne Bingle, Helen M. Marriott, Elizabeth E. LeClair, Colin D. Bingle

**Affiliations:** 1 Academic Unit of Respiratory Medicine, Department of Infection and Immunity, University of Sheffield, Sheffield, S10 2JF UK; 2 Department of Biomolecular Sciences, Faculty of Applied Sciences, University Technology MARA, Shah Alam, Selangor, Malaysia; 3 Oral and Maxillofacial Pathology, Department of Clinical Dentistry, University of Sheffield, Sheffield, UK; 4 Department of Biological Sciences, DePaul University, Chicago, IL USA

**Keywords:** BPIFA1, BPIFB1, SPLUNC1, LPLUNC1, Mouse (C57BL6; C57BL6/129sv)

## Abstract

Despite being initially identified in mice, little is known about the sites of production of members of the BPI fold (BPIF) containing (PLUNC) family of putative innate defence proteins in this species. These proteins have largely been considered to be specificaly expressed in the respiratory tract, and we have recently shown that they exhibit differential expression in the epithelium of the proximal airways. In this study, we have used species-specific antibodies to systematically localize two members of this protein family; BPIFA1 (PLUNC/SPLUNC1) and BPIFB1 (LPLUNC1) in adult mice. In general, these proteins exhibit distinct and only partially overlapping localization. BPIFA1 is highly expressed in the respiratory epithelium and Bowman’s glands of the nasal passages, whereas BPIFB1 is present in small subset of goblet cells in the nasal passage and pharynx. BPIFB1 is also present in the serous glands in the proximal tongue where is co-localised with the salivary gland specific family member, BPIFA2E (parotid secretory protein) and also in glands of the soft palate. Both proteins exhibit limited expression outside of these regions. These results are consistent with the localization of the proteins seen in man. Knowledge of the complex expression patterns of BPIF proteins in these regions will allow the use of tractable mouse models of disease to dissect their function.

**Electronic supplementary material:**

The online version of this article (doi:10.1007/s00441-012-1490-9) contains supplementary material, which is available to authorized users.

## Background

Palate lung nasal epithelium clone (*PLUNC*) was first described in the nasal epithelium of the mouse embryo and the trachea/bronchi of adult mice (Weston et al. 1999). We cloned the human and mouse *PLUNC* genes (Bingle and Bingle. 2000; LeClair et al. 2001), and subsequently made the key observation that PLUNC belongs to a group of proteins that make up the largest branch of a lipid transfer protein family. This group includes phospholipid transfer protein (PLTP), cholesterol ester transfer protein (CETP), bactericidal permeability increasing protein (BPI) and LPS-binding protein (LBP) (Bingle and Craven 2002; Bingle and Craven 2004; Bingle et al. 2004). Structural similarity across the PLUNC/BPI family suggested that these proteins would function by binding lipid molecules (Beamer et al. 1997; Bingle and Craven 2004), and this led to the hypothesis that PLUNCs may share host defence functions with BPI and LBP (Bingle and Craven. 2002).

PLUNC proteins are encoded by genes in a single locus on human chromosome 20q11, and conserved loci are found in all mammals. PLUNC proteins encoded by these genes were originally grouped into short (SPLUNC1 etc.) and long (LPLUNC1 etc.) proteins on the basis of structural homology to the domains of BPI, with SPLUNCs having structural similarity to the N-terminal domain of BPI, and LPLUNCs having structural similarity to both domains (Bingle and Craven. 2002). Due to the increasing complexity of this gene family and conflicting gene nomenclature, a new, comprehensive nomenclature has recently been developed. Within this framework, all family members have been renamed using the root symbol BPIF# for *“BPI fold containing”.* Family members that contain a single domain have the designation BPIFA (so that SPLUNC1/PLUNC becomes BPIFA1), and those containing two domains have the designation BPIFB (so that LPLUNC1 becomes BPIFB1) (Bingle et al. 2011b).

BPIF genes are amongst the most rapidly evolving mammalian genes and there is significant interspecies diversity in the wider family, particularly within the *BPIFA* branch (Bingle et al. 2004; Bingle et al. 2011a). The interspecies diversity and rapid evolution support a role for these proteins in host defence, although compelling functional data is only just starting to emerge (Wright et al. 2010; Gally et al. 2011; Lukinskiene et al. 2011). BPIFA1 has also been shown to have a surfactant-like function (Gakhar et al. 2010), and also to be involved in the activation of the sodium channel, ENaC (Garcia-Caballero et al. 2009). The sites of expression of BPIF proteins are not well-characterised, and indeed many of the genes have never been studied. However, existing data indicate that these genes are predominantly expressed in locations where innate defence is a major requirement, namely in the nasal, tracheal, and bronchial passages, as well as in major salivary glands and minor mucosal glands of the oral cavity (reviewed in Bingle and Bingle. 2011). Expression of these genes outside of these regions has also been shown, including *Bpifa1* in the mouse thymus (LeClair et al. 2001) and *Bpifb1* in the gastrointestinal tract (LeClair et al. 2001; Hou et al 2004). The prototypic two BPI-domain containing protein, BPIFB1 (LPLUNC1) and the founding family member BPIFA1 (SPLUNC1/PLUNC) are highly expressed in the upper respiratory tract (reviewed in Bingle and Bingle 2011). These two family members are present in all mammals (Bingle, Seal and Craven. 2011), and both are also readily detectable in human nasal secretions, bronchoalveolar lavage (BAL), and sputum (Casado et al. 2005; Wu et al. 2005; Nicholas et al. 2006).

Despite initially being cloned in mice, these genes are actually less well-described in this species. *Bpifa1* is expressed in the respiratory epithelium of the nasal passages, as well as in the large airways (Weston, et al 1999; LeClair et al. 2001; Genter et al. 2003; LeClair et al. 2004). Similar nasal expression domains have been shown in the rat (Sung et al. 2002). Our observations on the expression of *Bpifb1* in the epithelium and minor glands of the dorsal tongue and in the large airways, along with the report of expression in the early embryo, remain the only studies of expression of this gene in mice (Hou et al. 2004; LeClair et al. 2004). In contrast, there is extensive information concerning the localisation and expression of mouse and rat BPIFA2E (previously known as parotid secretory protein/psp) in the major salivary glands (reviewed in Ball et al. 2003). Indeed, this protein is the best studied member of this family.

In the present study, we have used mouse specific antibodies to BPIFA1 and BPIFB1 to carry out an extensive analysis of protein localisation in the nasal and oral cavities of adult mice. Knowledge of the patterns of BPIF proteins in these regions will be useful to inform studies of mouse models of disease to dissect their function.

## Materials and methods

### Generation of mouse specific BPIFA1 and BPIFB1 antibodies

Affinity-purified, peptide–specific, polyclonal antibodies against murine BPIFA1 and BPIFB1 were generated in rabbits using established methods (Eurogentec, Seraing, Belgium). For each protein, two anti-peptide antibodies were produced. For BPIFA1 antibody “A” the peptide sequence was: 189-199: (AVKDNQGRIHL), and for BPIFA1 antibody “B” the peptide sequence was 31-46: (GPPLPLNQGPPLPLNQ). This second region is absolutely unique for the mouse protein, as it is encoded by a mouse-specific insertion into exon 2. For BPIFB1 antibody “A”, the peptide sequence was: 140-150: (RVERSKSGPAH) located in the first BPI domain of BPIFB1, and for BPIFB1 antibody “B” the peptide sequence was 268-279: (LMETTPDRAPFS) in the second BPI domain. These peptides were chosen so as to have minimal sequence conservation between man and mouse, and no similarity with other members of the BPIF family. Bioinfomatic analysis also showed that the epitopes had no significant sequence identity with any other mouse proteins. Each individual peptide was used to affinity purify the final antibodies. We used the same methods to generate an antibody against mouse BPIFA2E (parotid secretory protein) using the peptide sequence CSSNTDKISISLLGRR corresponding to amino acids 161-176 (Khovidhunkit et al. 2005). (Eurogentec, Seraing, Belgium). This antibody was used as a comparator in studies of salivary glands as this protein is well-studied in these regions (Ball et al 2003).

### Sample collection

Mice strains C57BL6 and C57BL6/129sv were used in this study. They were housed in the DePaul University and the University of Sheffield animal care facilities, and were allowed access to food and water ad libitum. All studies were performed with full local ethics approval. We collected an extensive range of tissues from mice aged from 6 to 16 weeks, with some tissues being collected following perfusion fixation and all samples being fixed in cold 4 % paraformaldehyde. Where required, decalcification was performed using formic acid. Fixed tissues were embedded in paraffin according to standard protocols.

### Immunohistochemistry

Immunostaining was performed on 4-μm paraffin sections mounted onto glass slides (SuperFrost Plus, VWR international, Belgium). Slides were dried in the oven for 90 minutes to ensure the sections were firmly attached. All reactions followed standard protocols. Sections were deparaffinized and rehydrated. Endogenous peroxidase activity was blocked by quenching the sections in 3 % H_2_O_2_ in methanol for 20 minutes. The sections were then rinsed in PBS, and where necessary (for antibodies to BPIFB1) antigen retrieval was performed using tri-sodium citrate buffer for 8 minutes in a microwave oven followed by rinsing in PBS. Sections were incubated in 100 % normal goat serum for 30 minutes at room temperature in a humidified chamber. The serum was drained and replaced with primary antibody diluted in 100 % normal goat serum. Optimal antibody dilutions were determined empirically, and the final dilutions used were 1:750 BPIFA1, 1:750: BPIFB1, 1:500 PBIFA2E (Psp) and 1:500 MUC5B (H-300: sc-20119, Santa Cruz). Primary antibodies were left on the sections overnight at 4 °C in a humidified chamber. Rabbit IgG (DAKO) was used in place of the primary antibody as a negative control on representative slides, and a duplicate section was always used as a no primary antibody control. A Vectastain Elite ABC kit (Vector Laboratories) containing a goat anti-rabbit biotin-labelled secondary antibody was used according to the manufacturer’s instructions. Peroxidase enzymatic development was performed using a Vector NovaRed substrate kit, resulting in red staining in positive cells. Sections were counterstained with haematoxylin, dehydrated to xylene, and mounted in DPX. On occasions, Alcian blue staining of acidic mucus was performed using standard histological protocols prior to staining with BPIFA1.

## Results

Although *Bpifa1* (*plunc*/*splunc1*) was initially cloned from mice, limited published information exists on the sites of expression in this species. To inform our choice of tissue samples, we interrogated mouse tissue expression datasets (Su et al. 2004) on the BioGPS portal, http://biogps.org (Wu et al. 2009) for expression of *bpifa1* and *bpifb1*. This analysis suggested that both genes have very limited expression domains and are broadly found in similar regions of the nasal cavities, including the nasal septum, the nasal septal organ, the vomeronasal organ (VNO), and the olfactory epithelium, as well as in the trachea and to a lesser extent the lung (Sup Fig. 1). These data also indicated that *bpifb1* is expressed in the stomach.

### BPIFA1 and BPIFB1 are differentially expressed in the nasopharyngeal regions

Because of the low level of sequence similarity between human and mouse BPIF proteins, we generated mouse-specific antibodies against BPIFA1 and BPIFB1 for use in our studies. Both BPIFA1 antibodies and one BPIFB1 antibody (Antibody “B”) worked well for immunohistochemistry (IHC), and crucially they failed to show specific staining in tissue samples from mice deficient in BPIFA1 and BPIFB1 (Sup Fig. 2). We have also recently used these antibodies for the identification of the proteins in mouse bronchoalveolar lavage (BAL) fluid, and to show the differential localisation of the proteins in the respiratory tract (Bingle et al. 2012).

**Fig. 1:**
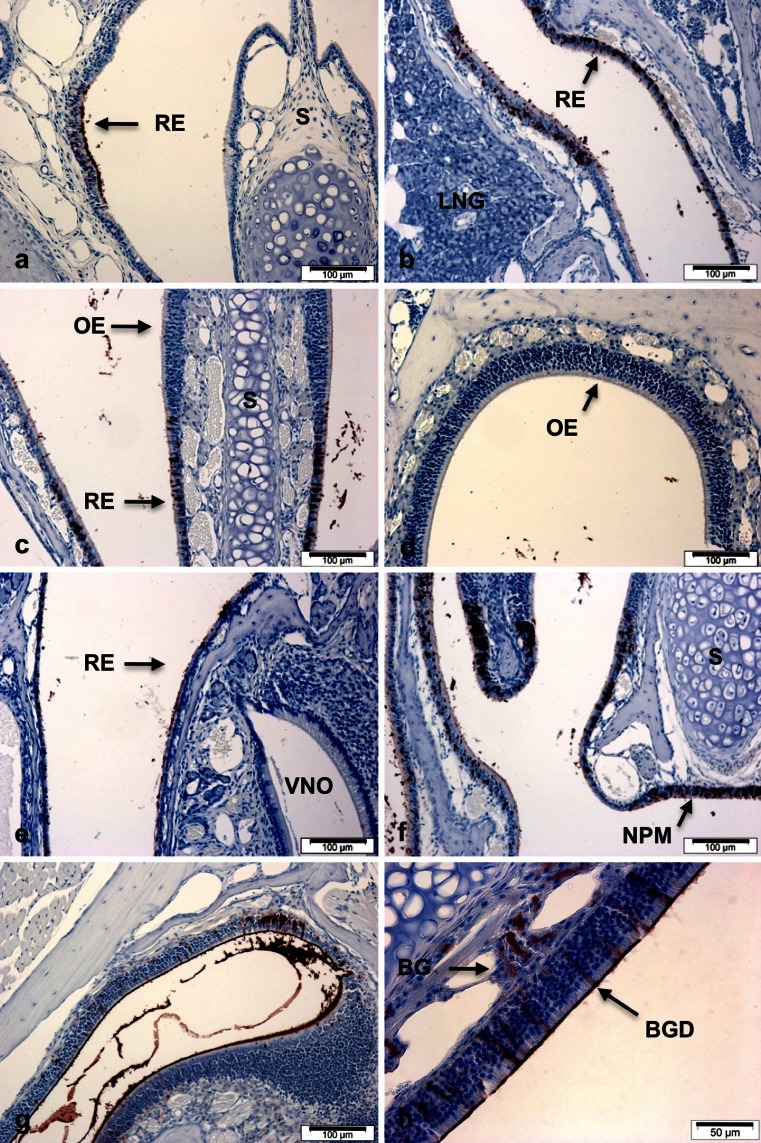
Distribution of BPILA1 in adult mouse nasal passages. Immunohistochemistry for BPIFA1 was performed as described in Materials and methods section. Sections show staining in samples of nasopharyngeal tissues sectioned it a proximal to distal manner. *RE* = respiratory epithelium, *S* = septum, *LNG* = lateral nasal gland, *OE* = olfactory epithelium, *VNO* = vomeronasal organ, *NPM* = nasopharyngeal meatus, *BG* = Bowman’s gland, *BGD* = Bowman’s gland duct

Given the original isolation of *bpifa1* from the mouse nasal septum, we surveyed the localisation of BPIFA1 in the adult head using a series of coronal sections starting from the snout. Within the anterior nasal cavity, most of the surface is covered with squamous epithelium, and in this region BPIFA1 is present in the patches of respiratory epithelium (Fig. 1a) on the lateral surface. The transitional epithelium overlaying the nasal septum was negative. In more caudal/posterior sections there was always a very clear demarcation between staining in the respiratory epithelium (Fig. 1b–f) and complete lack of staining in the transitional epithelium and olfactory epithelium of these regions. No staining was seen in the lateral nasal gland (Fig. 1b), vomeronasal organ (VNO), or associated minor septal glands (Fig. 1e). In more distal regions of olfactory epithelium associated with the dorsal medial meatus and the dorsal lateral meatus, staining of some Bowman’s glands and Bowman’s gland ducts was seen (Fig. 1g, h). In this region, BPIFA1 was found to be associated with the cell surface and in the mucous of the nasal passages. Closer examination of the respiratory epithelium from the nasal passages suggests that the BPIFA1 was present in non-ciliated cells.

**Fig. 2:**
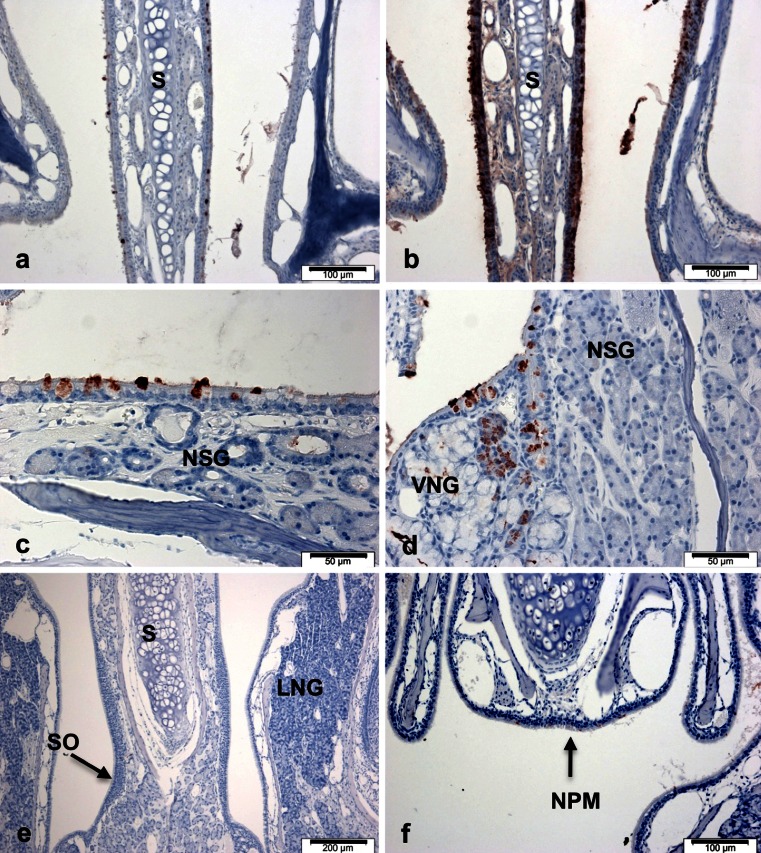
Distribution of BPILB1 in adult mouse nasal passages. Immunohistochemistry for BPIFB1 was performed as described in materials and methods section. Sections show staining in samples of proximal nasal septum (**a**), and more distal regions of the nasopharynx (**c**-**f**) Staining of BPIFA1 in the proximal nasal septum is shown for comparison (**b**) with **a**. *S* = septum, *NSG* = nasal septal gland, *VNG* = vomeronasal gland, *SO* = septal organ, *LNG* = lateral nasal gland, *NPM* = nasopharyngeal meatus

When we examined the localisation of BPIFB1 in similar sections, there were clear differences in intensity of staining and cellular localisation. Specifically, BPIFB1 expression is much more restricted. In anterior sections, BPIFB1 was limited to a population of goblet cells on the lateral surface of the nasal septum (Fig. 2a), whereas BPIFA1 staining was much stronger in the respiratory epithelium (Fig. 2b). Expression of BPIFB1 in goblet cells was confirmed at higher magnification of sections of the proximal septum (Fig. 2c); however, the nasal septal glands lacked expression. Goblet cells around and within the vomeronasal gland ducts and serous acinar cells within the gland were also positive for staining. However, the mucous portions of the gland were negative (Fig. 2d). In more posterior sections of the snout, there was no staining of BPIFB1 in the olfactory epithelium and Bowman’s glands (not shown), nor in the lateral nasal glands and the septal organ (Fig. 2e). In marked contrast to the situation seen with BPIFA1, only a very small number of cells in the respiratory epithelium of the nasopharyngeal meatus stained with BPIFB1 (Fig. 2f). In other sections, we did not observe staining for either protein in lacrimal glands, Harderian glands, Zymbal glands, or sebaceous glands (results not shown).

### BPIFB1 is predominantly localised to the serous cells of the minor glands of the proximal tongue and the soft palate

**Fig. 3:**
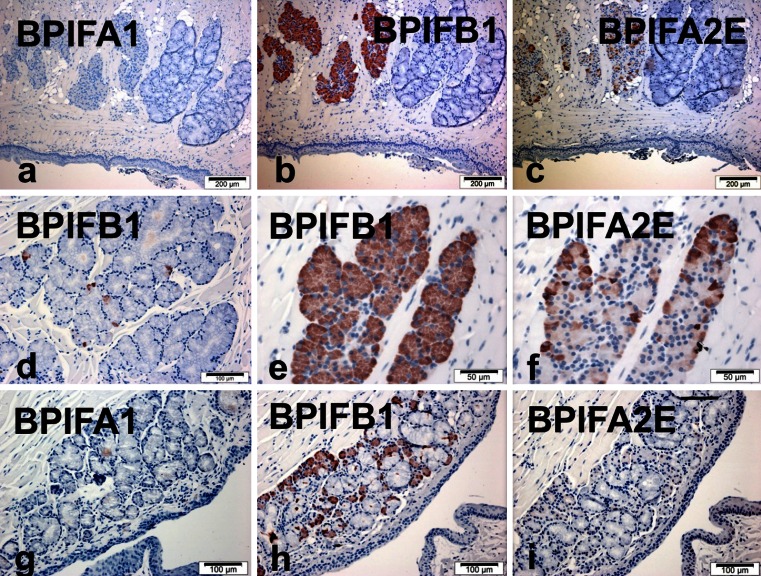
Distribution of BPIF proteins in the minor glands of the proximal mouse tongue and soft palate. Immunohistochemistry for BPIFA1 (**a**, **g**), BPIFB1 (**b**, **d**, **e**, **h**) and BPIFA2E (**c**, **f**, **i**) was performed in serial sections as described in materials and methods section. Sections show staining of the proximal tongue (**a**-**f**) and seromucus glands in the roof of the soft palate (**g**-**h**)

Analysis of sections of proximal tongue revealed that BPIFA1 staining was always negative (Fig. 3a). In contrast, the minor serous glands stained strongly with BPIFB1 (Fig. 3b, e), whereas the mucous glands were predominantly negative (Fig. 3b, d). Higher power views of the mucous glands did on occasion show a small number of BPIFB1-positive cells within the mucous acini (Fig. 3d). A similar, but less homogenous staining pattern was seen with BPIFA2E (Fig. 3c, f) but the protein was never detected in the mucous glands. BPIFB1 was also observed in the serous component of the seromucous glands overlying the soft palate (Fig. 3h), whereas staining for BPIFA1 and BPIFA2E staining was not observed (Fig. 3g, i). The surface epithelium of the proximal tongue and the soft palate was negative for all three proteins.

### BPIFA1 and BPIFB1 are differentially expressed in larynx

**Fig. 4:**
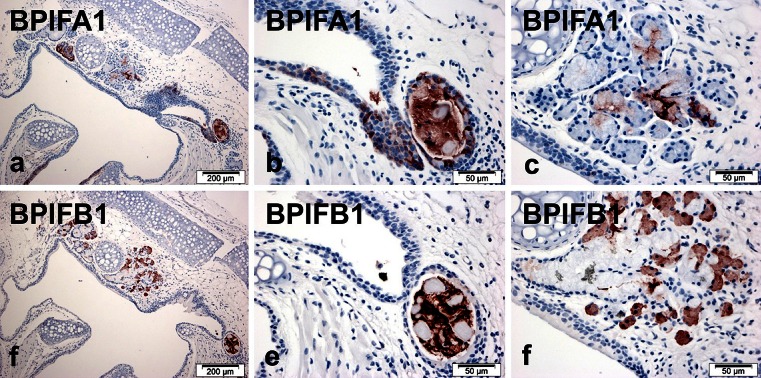
Distribution of BPIF proteins in the adult mouse larynx. Immunohistochemistry for BPIFA1 (**a**-**c**) and BPIFB1 (**d**-**f**) was performed in serial sections as described in materials and methods section. Sections show staining in regions of the epithelium and associated glands

Examination of the larynx showed that both proteins were present in the submucosal glands and ducts associated with this region (Fig. 4), but they appeared in different cells. Strong BPIFA1 was seen in cells of the ducts and within the luminal contents (Fig. 4a, b), with weaker staining in some of the serous and mucous cells of the glands themselves (Fig. 4c). The squamous epithelium lining the larynx was negative for staining; however, where this epithelium transitioned into respiratory epithelium at the opening of the trachea, strong staining was seen (Fig. 4a). In contrast, BPIFB1 did not stain the ductal epithelium but rather the luminal contents (Fig. 4d, e) as well as the serous cells of the submucosal glands, (Fig. 4f). Again, the squamous epithelium was negative for staining. The submucosal glands surrounding the larynx appear to represent the only site in the mouse where these two proteins are co-expressed within the same gland.

### BPIFA1 and BPIFB1 exhibit limited expression in other tissues

**Fig. 5:**
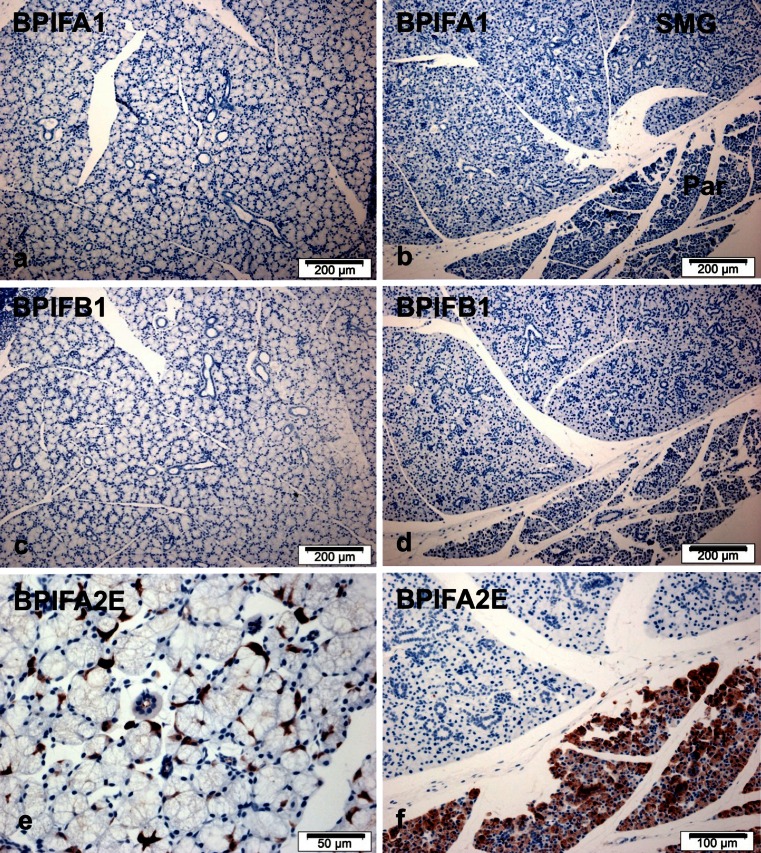
BPIFA1 and BPIFB1 are not expressed in the major salivary glands of the mouse. Immunohistochemistry for BPIFA1 (**a**, **b**), BPIFB1 (**c**, **d**) and BPIFA2E (**e**, **f**) was performed in serial sections of sublingual gland (**a**, **c**, **e**) and parotid and submandibular glands (**b**, **d**, **f**) as described in materials and methods section. *SGM* – submandibular gland; *Par* – parotid gland

Given our previous data on expression of human BPIF proteins in the major salivary glands (Bingle et al. 2005; Bingle et al. 2007; Bingle et al. 2010), we next studied samples of the three major salivary glands: the sublingual gland, the submandibular gland, and the parotid gland. In contrast to the situation seen in man, neither BPIFA1 (Fig. 5a, b) or BPIFB1 (Fig. 5c, d), were detected in any of these glands. Abundant BPIFA2E was detected in the serous demilunes of the sublingual gland (Fig. 5e) and throughout the serous acini of the parotid gland (Fig. 5f), but not in the submandibular gland.

We systematically analysed additional adult mouse tissues for expression of both proteins, to confirm that they were both predominantly expressed in the oral, nasopharyngeal, and respiratory tissues. Specifically, we did not see expression in the kidney (Sup Fig. 3a, b), heart, liver, pancreas, spleen, testes, or ovaries (data not shown). We focused on the GI tract, given the previous data showing *bpifb1* expression in the stomach and neonatal GI tract (LeClair et al. 2004; Hou et al. 2004) and the recent reports of expression of human proteins in this region (Sentani et al. 2008: Shin et al. 2011). A survey of sections from the oesophagus to the rectum revealed that there was a population of BPIFB1-positive cells in the fundus of the gastric glands (Sup Fig. 3d). BPIFA1 was not seen in this location (Sup Fig. 3c) and neither protein was seen in any other regions of the GI tract, including the ileum and colon (Sup Fig. 3e, f and g, h respectively).

## Discussion

Despite the fact that both *bpifa1* and *bpifb1* were originally cloned from mouse heads over 10 years ago (*bpifa1* from nasal tissue and *bpifb1* from proximal tongue), information on their expression domains is quite limited and is exclusively based on RNA expression. One of the most interesting observations on members of the wider BPIF-family is that they share relatively limited sequence similarity both within and between species (Bingle et al. 2004: Bingle, Bingle and Craven. 2011). This key observation has allowed us to generate a series of unique species-specific antibodies to study BPIFA1 and BPIFB1 localisation in mice. Our study of these two proteins in adult mice has allowed us to gain a fuller understanding of the sites of production, and has confirmed that both proteins are largely confined to regions of the head and upper respiratory tract. We have also recently reported that the two proteins are expressed in distinct epithelial cell populations in the airway. BPIFA1 is expressed in non-ciliated cells of the upper airway and the bronchial passages, whereas BPIFB1 stains a limited population of goblet cells in the upper airways. Neither protein is expressed in the small airways or in peripheral lung tissue (Bingle et al 2012). In this report, we described the systematic localisation of BPIFA1 or BPIFB1 in the mouse, a model that will be of value in understanding the biological function of these proteins.

*Bpifa1* was originally identified in developing nasal passages and the upper respiratory tract of adult mice (Weston et al. 1999). Our immunohistochemical analysis confirms that these are the major sites of expression. The most intense staining for BPIFA1 is in regions of the nasal passages, and within these regions the protein is localised to epithelial cells within the respiratory epithelium. This is also consistent with previous studies of rat *bpifa1* (Sung et al. 2002). The protein is not found in the squamous, transitional, or olfactory epithelium. The protein is associated with mucoid secretions of the nasal turbinates as well as with the ciliated surface overlying the olfactory epithelium. It seems likely that BPIFA1 is secreted by Bowman’s glands, as both the glands and their associated ducts stain strongly for BPIFA1. The protein does not seem to be produced by any of the other multiple minor glands associated with the nasal passages, nor does it appear to be produced by the vomeronasal or the nasal septal organs. Consistent with this localisation, the protein is readily detected in nasal washes by Western blotting (results not shown), and the data suggest that the protein is secreted into the mucosal lining fluids in these locations where it likely plays a host defence role.

Mouse *bpifb1* was originally cloned (NCBI accession number U46068) from minor glands of the proximal tongue and our in situ hybridization studies confirmed this, along with expression in the epithelium of the upper airways (LeClair et al. 2004). The present study further confirms that the protein is found in the serous portions of minor glands of the proximal tongue. Interestingly, here it appears to be expressed in the same cells as the well-studied rodent family member, BPIFA2E (formally known as parotid secretory protein) (Ball et al. 2003). Such co-localisation is the exception rather than the rule, as BPIFB1 is not produced in the sublingual and parotid glands, which strongly express BPIFA2E. Conversely, BPIFB1 is expressed in the serous demilunes of the seromucus glands of the soft palate, where BPIFA2E is not expressed. In the submucosal glands of the larynx (and trachea), BPIFB1 and BPIFA1 are present in different cellular compartments. BPIFB1 is found in the serous cells, whereas BPIFA1 is present in mucous cells. This distribution mirrors that seen in human airway submucosal glands and other minor mucosal glands (Bingle et al. 2005; Bingle et al. 2010). It remains to be determined why members of this family exhibit such a precise spatial expression pattern within these glands, given that secretory products from the serous and mucous cells ultimately end up being secreted into the same biological fluids. Interestingly, there do appear to be interspecies differences in the distribution of BPIF proteins in the major salivary glands. BPIFA1 and BPIFB1 are expressed in human major salivary glands (Bingle et al. 2005; Vargas et al 2009; Bingle et al. 2010), and are readily detected in saliva (Ramachandran et al. 2006; Walz et al. 2006; Siqueira et al. 2008), but our data shows that they are absent, or lowly expressed, in adult mouse salivary glands. Furthermore, in mouse, BPIFA2E (parotid secretory protein) is a major component of saliva accounting for 20-30 % of salivary protein, whereas levels of the closest human orthologue of the protein in man, BPIFA2, are much less.

In addition to being present in the serous compartment of tongue minor glands, BPIFB1 is also present in a subset of what appear to be goblet cells within the nasopharynx This is particularly evident in sections through the nasal cavity and within the nasopharynx (Fig. 2). As we have previously reported for the human protein, not all goblet cells in these regions produce the protein (Sup Fig. 4), and goblet cells in other locations, including the GI tract (Sup Fig 3) and the conjunctiva (data not shown), do not stain for BPIFB1.

Outside of the head, neck, and upper respiratory tract, it appears that both of these proteins have limited expression in adult mouse tissues. Mouse *bpifa1* expression was originally reported in the adult mouse heart (Weston et al. 1999), whilst other studies have suggested that the gene is expressed in models of cardiomyopathy (Bekeredjian et al. 2010). We have been unable to detect the protein in normal adult heart tissues by immunohistochemistry, suggesting that the protein is either absent from this tissue or is produced at levels below the limit of detection by IHC. Human *BPIFA1* expression has also been reported in the stomach, colon, and kidney, leading to the suggestion that it plays a role in the regulation of processing of the sodium channel, ENaC in these location (Garcia-Caballero et al. 2009). We were unable to detect the protein in mouse kidney, nor in any portions of the mouse GI tract from the stomach to the rectum. This may reflect a true difference between species, or again may be due to the fact that expression is very low in these tissues, and below the detection limit of the IHC technique.

We previously reported that *bpifb1* was expressed in the prenatal GI tract (LeClair et al. 2004). Expression has also been reported in the adult mouse stomach (Hou et al. 2004; Kallio et al. 2010) as well as in the developing wallaby stomach (Kwek et al. 2009). Our protein studies support these observations, showing that BPIFB1 is present in a population of cells within the gastric fundus. We were unable to detect the protein in any other regions of the GI tract, suggesting that it is not a significant product of the gastrointestinal mucosa. As far as we are aware, the protein has not previously been reported in the mouse GI tract, but we have recently shown that the protein is present in a population of Paneth cells in the human duodenum (Shin et al. 2011), where it has been shown to decrease pro-inflammatory responses to *V. cholerae* and *E. coli* LPS.

In summary, we have shown that the two most abundantly expressed members of the mouse BPIF protein family, BPIFA1 and BPIFB1, are localised to a number of epithelial cell types and minor mucosal glands associated with the nose, mouth, and respiratory tract. BPIFA1 is most strongly expressed in the nasal respiratory epithelium, whereas BPIFB1 appears to be most strongly expressed in the minor glands of the proximal tongue. There is limited expression of BPIFB1 in a population of goblet cells in the nasopharynx. As is the case in man, the two proteins are produced by different epithelial cell types, and may play host defence roles in the mucosal fluids in these locations. Understanding where these proteins are produced in the mouse will be of benefit to future functional studies on these and other members of the BPIF family.

## Electronic supplementary material

Below is the link to the electronic supplementary material.

Sup Fig. 1 *Bpifa1* and *Bpifb1* exhibit restricted sites of expression in adult mouse tissues Array data of Bpifa1 and Bpifb1 in adult tissues (Su et al. 2004) was recovered from the BioGPS portal as outlined in Materials and methods section (JPEG 397 kb)

Sup Fig. 2 BPIFA1 and BPIFB1 antibodies fail to detect proteins in tissues from mice deficient in the specific gene. Immunohistochemistry for BPIFA1 (**a**, **b**) and BPIFB1 (**c**,**d**) was performed on sections of nasopharynx from wt (**a**) and *bpifa1-/-* mice (**b**) and on proximal tongue from wt (**c**) and *bpifb1-/-* mice (**d**) as described in Materials and methods section. (JPEG 364 kb)

Sup Fig. 3 BPIFA1 is not expressed in the murine kidney and GI Tract whereas BPIFB1 exhibits limited expression in the glandular stomach. Immunohistochemistry for BPIFA1 (**a**, **c**, **e**, **g**) and BPIFB1 (**b**, **d**, **f**, **h**) was performed on sections of kidney (**a**, **b**), glandular stomach (**c**,**d**) ileum (**e**, **f**) and colon (**g**,**h**) as described in Materials and methods section. (JPEG 1294 kb)

Sup Fig. 4 Localisation of BPILB1 in a population of goblet cells in the adult mouse nasopharynx. Immunohistochemistry for BPIFA1, BPIFB1 and MUC5B was performed as described in Materials and methods section. Sections show staining in replicate samples from the nasopharynx. (JPEG 196 kb)
