# Supplementary figures and images for: Differential localisation of BPIFA1 (SPLUNC1) and BPIFB1 (LPLUNC1) in the nasal and oral cavities of mice
Source: Cell Tissue Res. 2012 Sep 18;350(3):455–64. doi: 10.1007/s00441-012-1490-9 (PMC3505551; doi:10.1007/s00441-012-1490-9)

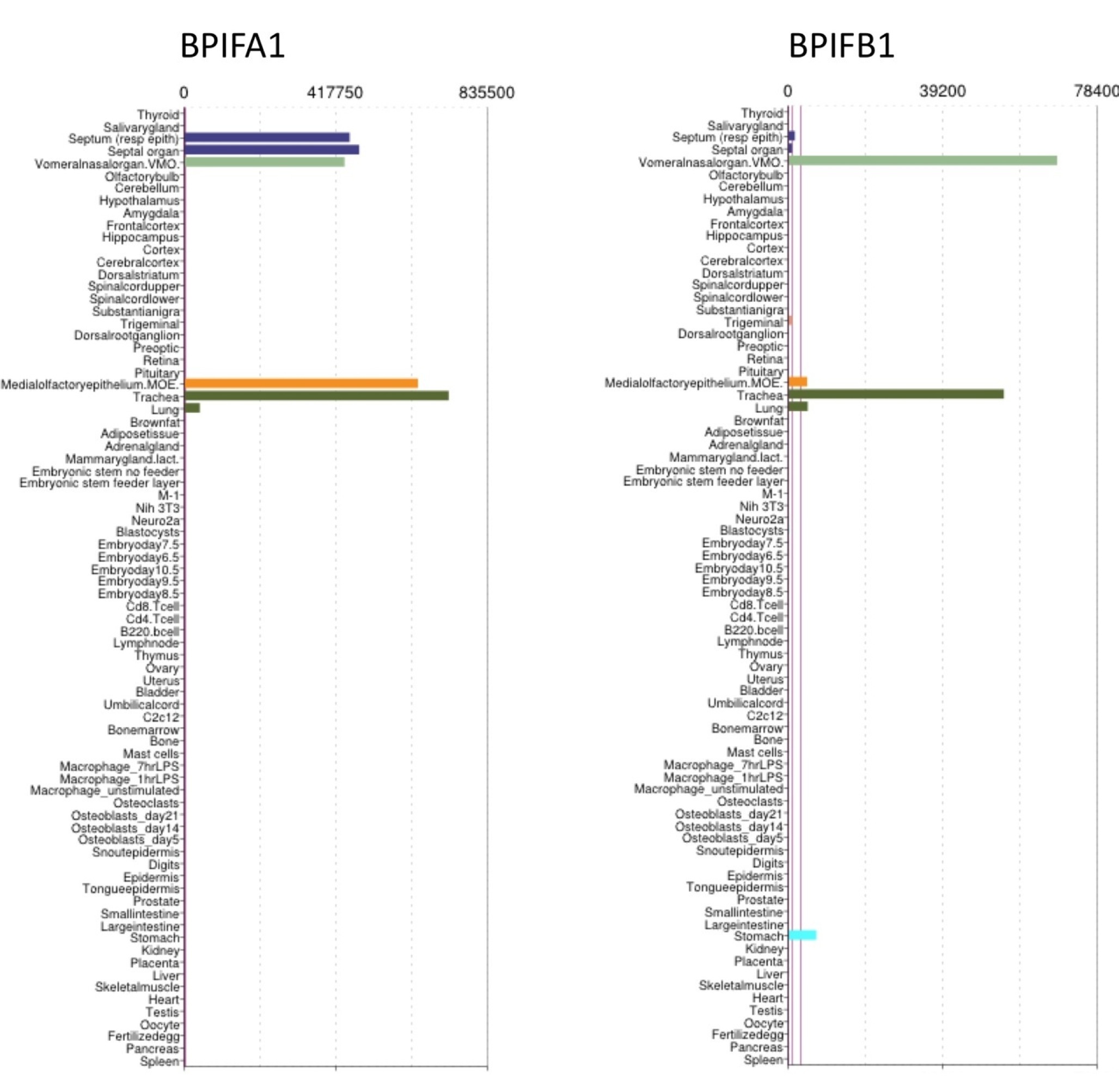

Supplement: Supplementary file 1 — Bpifa1 and Bpifb1 exhibit restricted sites of expression in adult mouse tissues Array data of Bpifa1 and Bpifb1 in adult tissues (Su et al. 2004) was recovered from the BioGPS portal as outlined in Materials and methods section (JPEG 397 kb) [file 441_2012_1490_MOESM1_ESM.jpg]

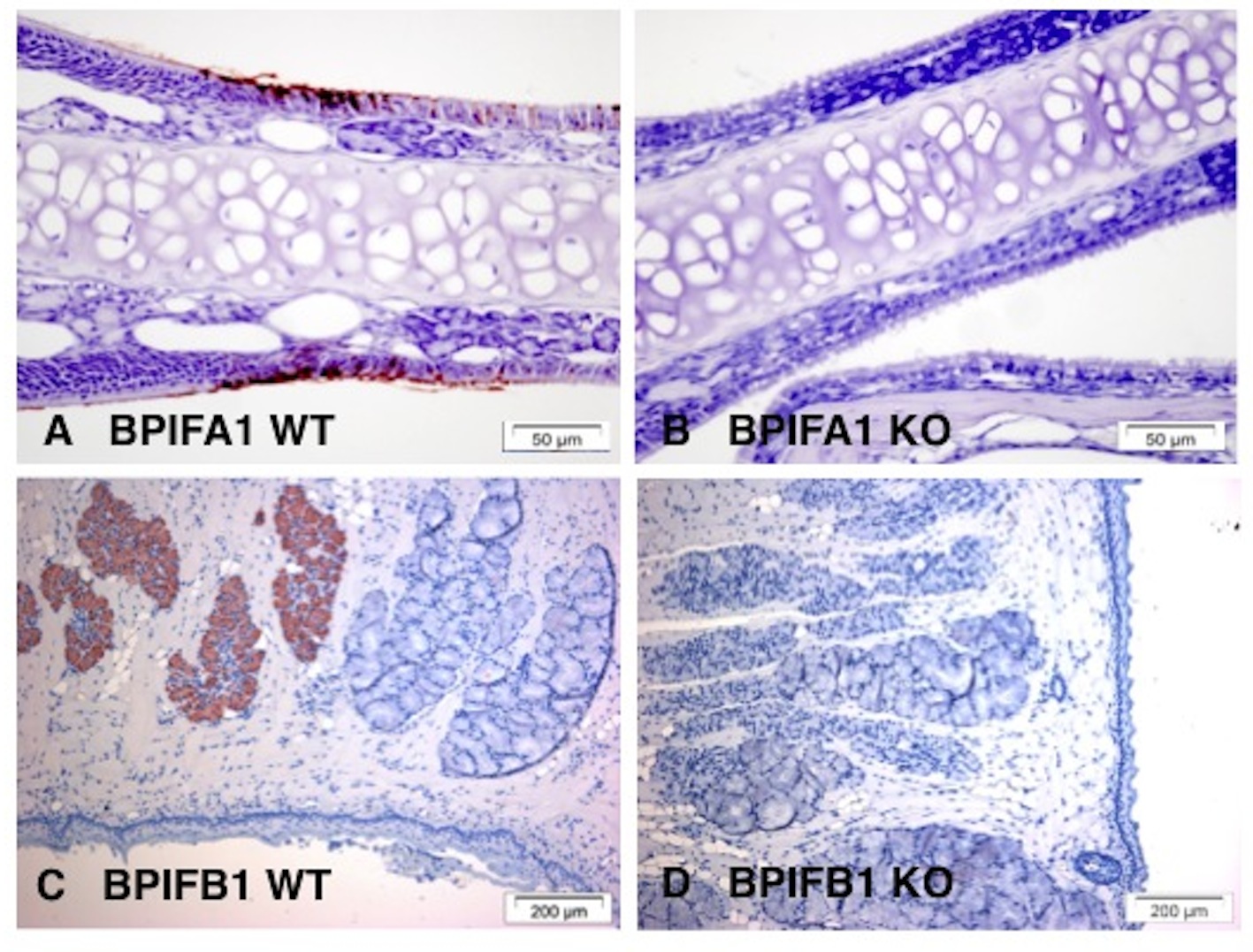

Supplement: Supplementary file 2 — BPIFA1 and BPIFB1 antibodies fail to detect proteins in tissues from mice deficient in the specific gene. Immunohistochemistry for BPIFA1 (a, b) and BPIFB1 (c,d) was performed on sections of nasopharynx from wt (a) and bpifa1-/- mice (b) and on proximal tongue from wt (c) and bpifb1-/- mice (d) as described in Materials and methods section. (JPEG 364 kb) [file 441_2012_1490_MOESM2_ESM.jpg]

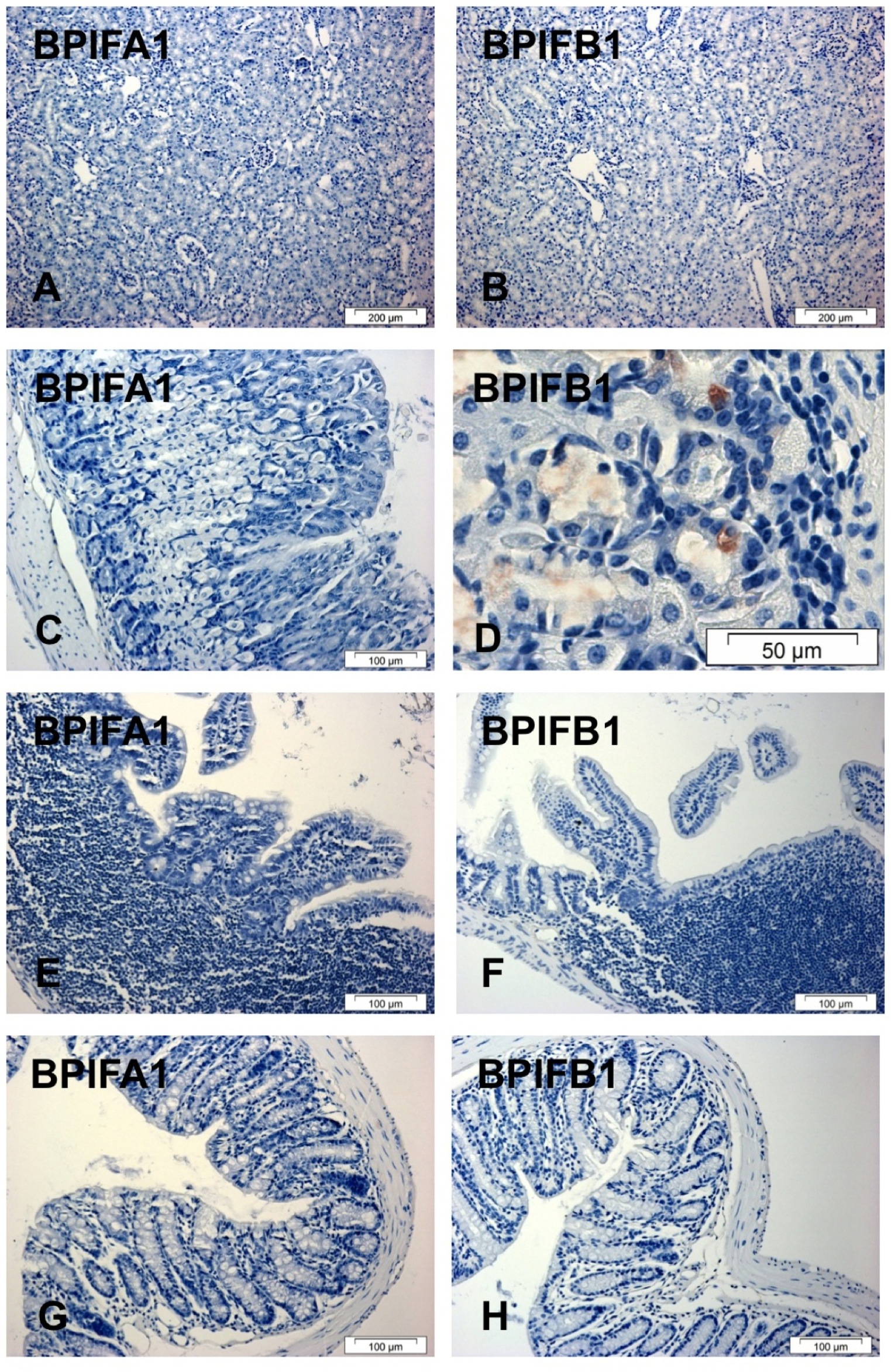

Supplement: Supplementary file 3 — BPIFA1 is not expressed in the murine kidney and GI Tract whereas BPIFB1 exhibits limited expression in the glandular stomach. Immunohistochemistry for BPIFA1 (a, c, e, g) and BPIFB1 (b, d, f, h) was performed on sections of kidney (a, b), glandular stomach (c,d) ileum (e, f) and colon (g,h) as described in Materials and methods section. (JPEG 1294 kb) [file 441_2012_1490_MOESM3_ESM.jpg]

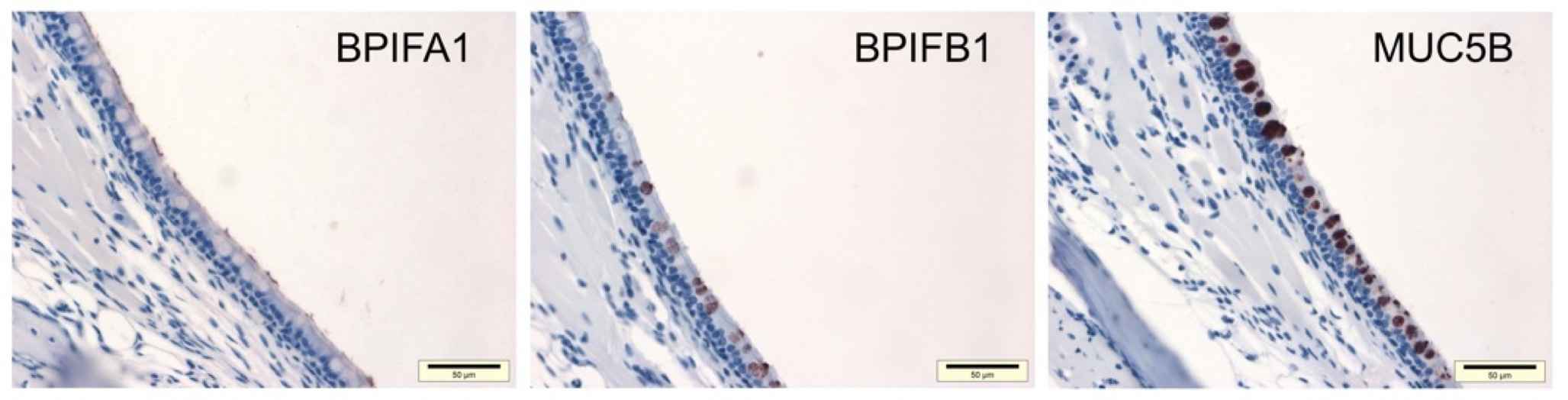

Supplement: Supplementary file 4 — Localisation of BPILB1 in a population of goblet cells in the adult mouse nasopharynx. Immunohistochemistry for BPIFA1, BPIFB1 and MUC5B was performed as described in Materials and methods section. Sections show staining in replicate samples from the nasopharynx. (JPEG 196 kb) [file 441_2012_1490_MOESM4_ESM.jpg]
